# Structural insight into activation of homoserine dehydrogenase from the archaeon *Sulfolobus tokodaii* via reduction

**DOI:** 10.1016/j.bbrep.2015.07.006

**Published:** 2015-07-15

**Authors:** Yoshihisa Tomonaga, Ryosuke Kaneko, Masaru Goto, Toshihisa Ohshima, Kazuaki Yoshimune

**Affiliations:** a Department of Applied Molecular Chemistry, Graduate School of Industrial Technology, Nihon University, 1-2-1, Izumichou, Narashino, Chiba 275-8575, Japan; b Department of Biomolecular Science, Graduate School of Science, Toho University, 2-2-1, Miyama, Funabashi, Chiba 274-8510, Japan; c Department of Biomedical Engineering, Osaka Institute of Technology, 5-16-1, Ohmiya, Asahi-ku, Osaka 535-8585, Japan

**Keywords:** HSD, homoserine dehydrogenase, Tris, tris(hydroxymethyl)aminomethane, PDB, Protein Data Bank, Tricine, *N*-[tris(hydroxymethyl)methyl]glycine, Bis-tris, 2,2-bis(hydroxymethyl)-2,2′,2″-nitrilotriethanol, HEPES, [4-(2-hydroxyethyl)-1-piperazinyl] ethanesulfonic acid, PEG, poly(ethylene glycol), DMSO, dimethyl sulfoxide, RMSD, root mean square deviation, LB medium, Luria-Bertani medium, IPTG, isopropyl 1-thio-β-d-galactoside, SDS-PAGE, sodium dodecyl sulfate-polyacrylamide gel electrophoresis, DTT, dithiothreitol, ORF, open reading frame, KPB, potassium phosphate buffer, mPMS, 1-methoxy-5-methyl-phenazinium methyl sulfate, Hyperthermophilic archaea, Homoserine dehydrogenase, Crystal structure, Disulfide bond, Activation

## Abstract

Homoserine dehydrogenase (HSD; 305 amino acid residues) catalyzes an NAD(P)-dependent reversible reaction between l-homoserine and aspartate 4-semialdehyde and is involved in the aspartate pathway. HSD from the hyperthermophilic archaeon *Sulfolobus tokodaii* was markedly activated (2.5-fold) by the addition of 0.8 mM dithiothreitol. The crystal structure of the homodimer indicated that the activation was caused by cleavage of the disulfide bond formed between two cysteine residues (C303) in the C-terminal regions of the two subunits.

## Highlights

• StHSD is activated by the addition of DTT.
• Structural analysis revealed a disulfide bond between the homodimer and the cleavage.
• Disulfide bond may decrease the activity of StHSD.

## Graphical abstract

**Figure:**
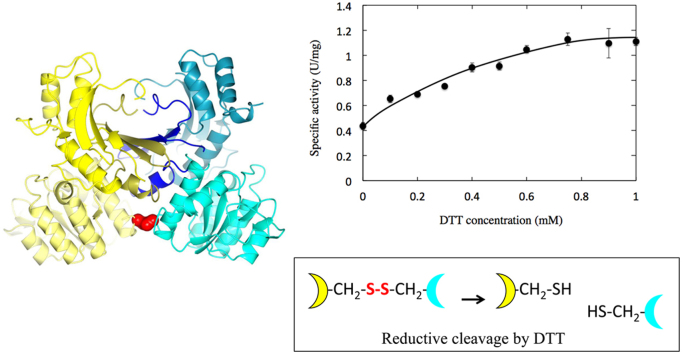

## 1 Introduction

HSD (EC 1.1.1.3) catalyzes the reversible NAD(P)-dependent oxidation of l-homoserine to l-aspartate 4-semialdehyde and NAD(P)H. The reverse reaction is responsible for the production of l-homoserine in the aspartate pathway, which produces four l-amino acids (lysine, threonine, cysteine, and methionine) as final products from l-aspartate [1]. Because its product l-homoserine is a precursor of l-threonine, l-cysteine, and l-methionine and its substrate l-aspartate 4-semialdehyde is a precursor of l-lysine, HSD plays a key regulatory role in the pathway. HSD is found in plants, fungi, and bacteria, but not in animals, indicating that HSD is a good target for new pesticides and antibiotics [2]. l-lysine is synthesized via the l-α-aminoadipate pathway in hyperthermophilic archaea and fungi, though the diaminopimelate pathway is utilized for lysine production in bacteria and plants [3]. HSD is often susceptible to feedback inhibition by end products in the aspartate pathway. HSD from *Corynebacterium glutamicum* is inhibited by both threonine and methionine [4], and the enzyme from *Thermus flavus* AT-62 is inhibited by cysteine [5]. The *Escherichia coli* enzyme is known to exhibit bifunctional activity as both an HSD and an aspartate kinase (EC 2.7.2.4) [6]. The bifunctional HSD catalyzes the first and third steps in the aspartate pathway and is found in bacteria and plants [7]. Usually, the bifunctional enzyme has more than 800 amino acid residues and is much larger than monofunctional HSD, which consists of approximately 300 amino acid residues. Based on their amino acid sequences, HSDs are divided into three groups: a short-chain group and two long-chain groups with extensions at the C-terminus [8]. The latter groups, which possess approximately 70-residue extensions at the C-terminus, are feedback inhibited by threonine or methionine. The biochemical and structural characteristics and regulation mechanisms of HSDs from bacteria, yeasts, plants, and animals have been extensively studied so far, but information on these enzymes from the third domain of life, i.e., the archaea, is extremely scanty. Thus, we screened the HSD gene homologs of archaeal strains in a genome data bank and found a hypothetical HSD gene from the acidophilic and hyperthermophilic archaeon *Sulfolobus tokodaii* (StHSD; 304 amino acids). In this study, we succeeded in overexpression of the gene product in *E. coli* and found that the purified enzyme is uniquely activated by a reducing agent DTT. In addition, based on structural analyses, we found a novel type of activation mechanism in which the cleavage of an intermolecular disulfide bond between the C-terminal regions of the two subunits is responsible for the enzyme activity.

## 2 Materials and methods

All reagents were purchased from Wako Pure Chemical Industries, unless otherwise noted. The expression plasmid for StHSD was constructed as follows. The hypothetical HSD gene from *S. tokodaii* NBRC100140^T^ was amplified using genomic DNA purchased from the National Institute of Technology and Evaluation. The primer set of 5′- CACCATGAAATTATTACTCTTTGGTTATGGAAATG-3′ and 5′- TCATAAGCAATCTCTCTTTAGAAGAATTAAATC-3′ was used for amplification of the ORF. The PCR product obtained was ligated into pET101 according to the manufacturer’s instructions for Champion™ pET Directional TOPO® expression kits (Invitrogen Life Technologies). The ORF in the resultant plasmid, pSTHSD, was confirmed to have the correct sequence. The *E. coli* BL21(DE3) strain was transformed with the pSTHSD plasmid, cultured at 310 K for 12 h in LB medium supplemented with ampicillin (50 μg/L), and then sonicated in 10 mM KPB (pH 7.0). StHSD was purified by single-column chromatography with DEAE-TOYOPEARL after heat treatment (343 K for 3 h) of the cell extract. The column was washed with 10 mM Tris–HCl (pH 8.0), and StHSD was eluted with 10 mM Tris–HCl (pH 8.0) containing 50 mM NaCl. The purified enzyme was dialyzed against 10 mM KPB (pH 7.0) and concentrated with an Amicon Ultra 10 K filter unit (Millipore). The homogeneity of the final StHSD preparation was confirmed by SDS-PAGE. The activation of StHSD by various concentrations of DTT was assayed using WST-1 (Dojindo Laboratories), which is reduced by NADH to form WST-1 formazan in the presence of the electron acceptor mPMS, in a reaction mixture consisting of 100 mM Tris–HCl (pH 8.0) and 1 mM DTT. The increase of the WST-1 formazan level was monitored at 438 nm. The StHSD activity was calculated using a molar coefficient of 37,000 at 438 nm for WST-1 formazan. The kinetic parameter was determined by measuring the increase in absorbance of the produced NADH at 340 nm (molar coefficient of 6,200) at 303 K in 100 mM Tris–HCl (pH 8.0). The oxidized form was prepared by the pre-treatment of StHSD with 0.1 mM potassium ferricyanide for 2 h. The reduced form was prepared by the pre-treatment with 1 mM DTT for 2 h. The protein concentration of the crude cell extract was measured by use of a Pierce^®^ BCA protein assay kit (Thermo Scientific Pierce).

Crystals of StHSD were grown at 285 K by the hanging-drop vapor diffusion method with 100 μL of reservoir solution [9.5% (w/v) PEG 3350, 19% (w/v) PEG 400, 0.19 M magnesium chloride, and 2.5% DMSO]. The droplets (pH 4.1) was prepared by mixing 1.5 μL of purified enzyme (5.9 mg/mL) and an equal amount of reservoir solution. The crystals of the reduced form were prepared by soaking crystals in the solution consisting of 3 μl of the reservoir solution and 1 μl of 200 mM DTT for 60 min prior to the first diffraction data collection. The crystals were flash-frozen using liquid nitrogen. Diffraction data were collected at beamlines at the Photon Factory, Tsukuba, Japan. The data sets were collected for the oxidized and reduced forms, respectively, at 95 K. All images were indexed and integrated using the program HKL2000 [9], and the data sets were phased with molecular replacement by using the program Phaser [10]. An initial phasing model was prepared by homology modeling using SWISS-MODEL [11]. The models were built using the program COOT [12] and refined using Refmac5 [13]. The two subunits in the asymmetric units were refined without no crystallographic symmetry restraints. Both the main and the side chains were clearly identified in the 2*Fo*–*Fc* electron density map, and the final difference Fourier maps did not contain any significant peaks. The programs RAMPAGE [14] and SFCHECK [15] in the CCP4 package were used for stereochemistry analysis of all models and for calculation of the RMSD as well as the average error by the Luzzati plot. The statistics for data collection and refinement are presented in Table 1. All figures illustrating these structures were prepared using the program CCP4mg [16]. The coordinates of the oxidized and reduced forms have been deposited in the PDB under entry number 4YDR and 5AVO, respectively.

**Table 1:** Data collection and refinement statistics.^a^

	Oxidized form	Reduced form
Data collection statistics		
Beamline	NE3A (PF-AR)	NE3A (PF-AR)
Wavelength (Å)	1.0000	1.0000
Resolution (Å)	50.0–1.60 (1.63–1.60)	50.0–1.80 (1.83–1.80)
No. of reflections (measured/unique)	345,770/74,576	119,944/47,593
*R*_merge_^b^ (%)	7.8 (29.5)	6.2 (26.1)
Completeness (%)	99.2 (100)	90.6 (94.8)
Multiplicity (%)	4.6 (4.9)	2.5 (2.4)
No. of crystals	1	1
Space group	P2_1_	P2_1_
Unit –cell constants		
*a* (Å)	57.401	57.818
*b* (Å)	79.472	78.909
*c* (Å)	65.899	65.814
*α* (deg)	90	90
*β* (deg)	107.16	105.79
*γ* (deg)	90	90
		
Refinement statistics		
Resolution range (Å)	28.5–1.60	50.0–1.80
No. of reflections	70,670	45,154
*R*_factor_ for 95% data^c^	0.197	0.211
Free *R*_factor_ for 5% data	0.228	0.247
No. of atoms		
Protein	4,576	4,500
Water	402	195
Sodium ion	1	0
RMSD from ideality		
Bond lengths (Å)	0.0078	0.0075
Bond angles (°)	1.2933	1.2445
Ramachandran analysis		
Favored (%)	96.5	97.3
Allowed (%)	3.5	2.7
Disallowed (%)	0	0

a Values in parentheses are statistics for the highest-resolution shell, whose range is 1.63–1.60  Å.

b *R*_merge_=Σ*hkl*Σ*i*|*I_hkl_*_,*i*_−〈*I_hkl_*〉|/Σ*hkl*Σ*iI_hkl,i,_* where *I*=observed intensity and 〈*I*〉=average intensity for multiple measurements.

c *R*_free_ was monitored with 5% of the reflection data excluded from the refinement.

## 3 Results and discussion

**Fig. 1:**
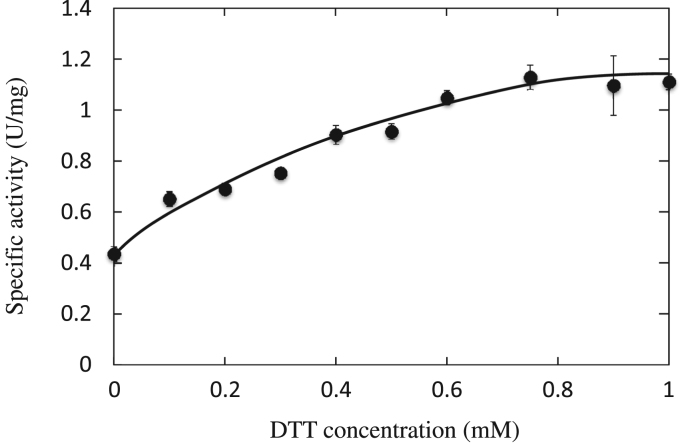
Activation of StHSD. The activity of StHSD was assayed in the presence of various concentrations of DTT. The error bars represent the standard deviations of the measurements. One unit of the enzyme was defined as the amount of the enzyme that produced 1 μmol of WST-1 formazan at 303 K in 1 min.

A comparative analysis of amino acid sequences of archaeal HSDs showed that StHSD is 304 amino acids long and that the gene is likely to start with a UUG initiation codon. This initiation codon usage is rarely found in *S. tokodaii* [17]. The hypothetical initiation codon (UUG) was mutated to AUG, and the resultant ORF was overproduced in *E. coli*. The crude enzyme showed a specific activity of 0.020 U/mg. The activity of purified StHSD increased up to 2.5-fold with the increase of DTT concentration (Fig. 1). There was no significant dose-dependent effect on the activity in the presence of more than 0.8 mM DTT. The addition of more than 0.1 mM DTT gave a small, nonenzymatic increase of the background absorbance. Such background absorbance can probably be attributed to the reduction of WST-1 by DTT to form WST-1 formazan. The comparison of kinetic parameters between the oxidized and reduced enzymes showed that the reduction increased the *K*_m_ and *V*_max_ values for l-homoserine, but gave relatively small effect on *K*_m_ and the *V*_max_ values for NAD (Table 2). Since StHSD (304 total amino acid residues) has only one cysteine residue (C303), near the C-terminus, we postulated that the activation of enzyme is induced by reductive cleavage of an interchain disulfide bond between the two subunits. Alteration of activity by reductive cleavage of an intrachain disulfide bond by DTT has been found in the bifunctional HSD from *E. coli* K-12 [18]. In this case, the addition of DTT activates the enzyme and increases the sensitivity of HSD to inhibition by the feedback modifier l-threonine.

**Table 2:** Kinetic parameters of the oxidized and the reduced forms.

Substrate	l-Homoserine			NAD	
	*K*_m_ (mM)		*V*_max_ (U/mg)	*K*_m_ (mM)	*V*_max_ (U/mg)
Oxidized form	0.21	0.81		0.31	1.0
Reduced form	0.54	1.8		0.33	1.3

The oxidized and the reduced forms were prepared by the pre-treatment of StHSD with 0.1 mM potassium ferricyanide or 1 mM DTT, respectively, for 2 h. The values are calculated based on Lineweaver–Burk plot. One unit of the enzyme was defined as the amount of the enzyme that produced 1 μmol of NADH at 323 K in 1 min.

**Fig. 2:**
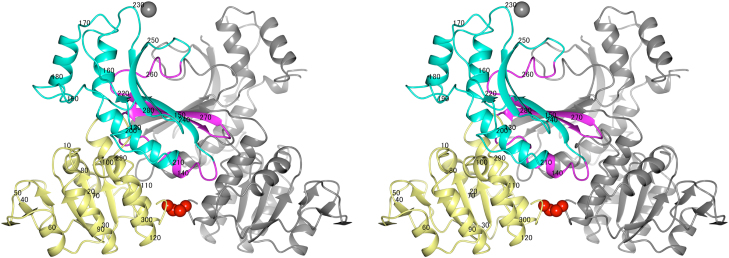
The structure of StHSD in the dimeric form. The nucleotide-binding region (residues 1–130 and 285–304), the dimerization region (residues 131–145 and 256–284), and the catalytic region (residues 146–255) are shown in yellow, magenta, and cyan, respectively. The other monomer is shown in gray. The disulfide bond between the two cysteine 303 residues is shown as red spheres. The sodium ion is shown as a gray sphere.

The crystal analysis of StHSD showed the overall structure of a homodimer with one disulfide bond in the asymmetric unit (Fig. 2). Each monomer of StHSD is composed of the nucleotide-binding region (amino acid residues 1–130 and 285–304), the dimerization region (residues 131–145 and 256–284), and the catalytic region (residues 146–255), as in the HSD from *Saccharomyces cerevisiae* (PDB entry 1EBU) [19]. The nucleotide-binding and catalytic regions are structurally well conserved among the equivalent regions of structurally characterized HSDs, although no significant structural homolog of the dimerization region was detected using the DALI database. A trigonal-bipyramidally coordinated sodium ion was located between the subunits of the oxidized form, though this location is apart from the equivalent position of the metal-binding site in HSD from *S. cerevisiae*[19].

**Fig. 3:**
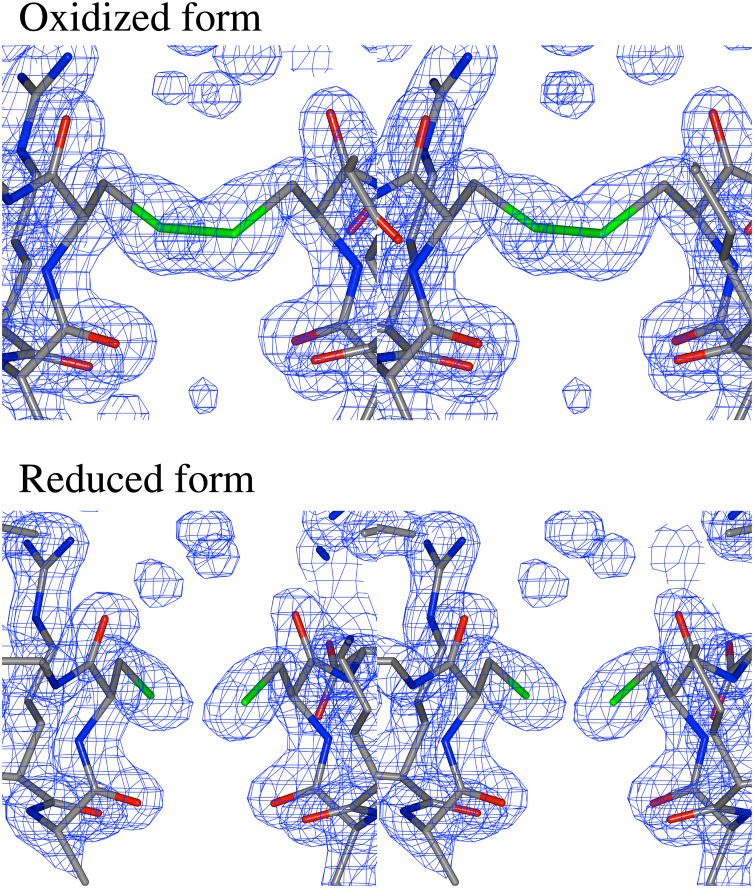
Cleavage of the disulfide bond. The disulfide bond between residues C303 was found without the oxidation by the addition of 0.1 mM potassium ferricyanide. The bond was cleavaged by the soaking in the presence of 50 mM DTT for 60 min prior to the first diffraction data collection.

In the crystal, an intermolecular disulfide bond was found to be formed between the cysteine 303 residues in the C-terminal regions of the monomers. In contrast, the cleavage of the disulfide bond in the reduced form of StHSD was observed (Fig. 3). The sulfur atoms of C303 in the reduced form refined at 1.80 Å resolution are separated by the distance of 2.9 Å which is significantly larger than the distance between the sulfur atoms of the disulfide bond (2.2 Å) in the oxidized form refined at 1.60 Å. Considering that the reducing reagent DTT activates StHSD by decreasing the *K*_m_ value for l-homoserine, the activation may be due to increased flexibility in the structure of the subunit, especially in the l-homoserine-binding site.

The C-terminal region of the long-chain group of HSDs from *C. glutamicum* is necessary for its allosteric inhibition by l-threonine [4]. Although StHSD has no extension at the C-terminus and belongs to the short-chain group, the activity of StHSD may be affected by the conformation of the C-terminal region. This hypothesis is consistent with the fact that the nucleotide-binding region including the C-terminal region of the HSD from *S. cerevisiae* slightly alters the active site geometry upon cofactor binding for the appearance of activity [19].

Among the known primary sequences of HSDs, the cysteine residue near the C-terminus is present in only the *Sulfolobus solfataricus* and *Sulfolobus islandicus* enzymes, indicating that these two archaeal HSDs may form intermolecular disulfide bonds and be activated by the addition of reducing agents, such as DTT, though the physiological implication of this observation remains unknown.
